# *Salmonella* in Free-Ranging Quokkas (*Setonix brachyurus*) from Rottnest Island and the Mainland of Western Australia

**DOI:** 10.3390/ani10040585

**Published:** 2020-03-31

**Authors:** Pedro Martínez-Pérez, Timothy H. Hyndman, Patricia A. Fleming

**Affiliations:** College of Science, Health, Engineering and Education of Murdoch University, Murdoch, Western Australia 6150, Australia; pedro_martinez-perez@np.edu.sg (P.M.-P.); t.fleming@murdoch.edu.au (P.A.F.)

**Keywords:** conservation, wildlife management, Cerro, enterica, salamae, diarizonae

## Abstract

**Simple Summary:**

Quokkas are small kangaroo-like mammals that are native to Western Australia. They live on Rottnest Island, where they are habituated to human contact, and they also live in forest environments on the mainland, where they are rarely in contact with people. It is known that quokkas can be infected with *Salmonella*. *Salmonella* is a type of bacterium that can result in diarrhoea and other signs of disease. It is known that animals can infect people with *Salmonella*. Many animals infected with *Salmonella* get sick after acquiring the infection. We collected faeces from 92 quokkas; 71 from Rottnest Island and 21 from mainland Western Australia. We detected *Salmonella* in almost half of the animals from Rottnest and only one animal from the mainland. We also examined the blood of these quokkas and found evidence that some of these infected quokkas had signs of mild disease that are consistent with a *Salmonella* infection. Our study revealed some new types of *Salmonella* in these quokkas.

**Abstract:**

*Salmonella* is a genus of Gram-negative, motile, and facultative anaerobic bacteria with a worldwide distribution that contaminates multiple substrates (vegetation, food, soil, and water) and inhabits the gastrointestinal tract of birds, reptiles, and mammals, including humans. Rottnest Island is a popular tourist destination and is abundantly inhabited by quokkas (*Setonix brachyurus*), a charismatic small wallaby. Current data on the association between *Salmonella* and quokkas on Rottnest Island are outdated by approximately 30 years. Additionally, previous studies on quokkas on this island and mainland Western Australia did not perform physical examinations or any diagnostic tests. The aim of the project was to assess the prevalence of *Salmonella* spp. in quokkas from Rottnest Island and mainland Western Australia and correlate the presence of the bacterium with the health of the animal. Ninety-two quokkas from Rottnest Island (n = 71) and populations on the mainland (n = 21) were screened for *Salmonella*, and a prevalence of 47.9% and 4.8%, respectively, was determined. A total of 16 serovars were identified from 37 isolates; five of these serovars had previously not been described in the quokka. *Salmonella* appeared to have an effect on the haematology and blood chemistry of quokkas on Rottnest Island consistent with subclinical salmonellosis. The health of Rottnest Island quokkas, and their potential impact on the health of the visitors to the island, should continue to be monitored carefully.

## 1. Introduction

*Salmonella* spp. are rod-shaped, Gram-negative, motile, and facultative anaerobes belonging to the family Enterobacteriaceae [1]. Under the White–Kauffmann–Le Minor scheme [2], there were 2579 serovars that belonged to two accepted species: *Salmonella enterica* (2557 serovars) and *Salmonella bongori* (22 serovars). These organisms have a worldwide distribution, contaminating vegetation, food, soil, and water, and inhabit the gastrointestinal tract of birds, reptiles, and mammals, including humans [3]. *Salmonella* can survive for up to nine months in the environment (e.g., soil, faecal material, food, and water) [4]. However, it is unclear whether *Salmonella* found in the environment represent free-living organisms or contaminants from animal faeces [5,6].

Clinical disease is commonly triggered by stressful events or conditions that could include sudden changes in diet, deprivation of feed, parturition, transportation, heat stress, and drought [3,7,8]. However, the severity of the disease is dependent on a combination of host factors (e.g., age, concurrent disease, and immune status of the animal) and *Salmonella* factors (e.g., strain and infective dose) [8,9]. Salmonellosis can vary from a subclinical carrier state to an acute endotoxemia and sepsis that is usually fatal.

Human salmonellosis has been linked in the past with wildlife and various pathways to infection have been studied, including direct contact, contamination of food by wildlife faeces, consumption of wildlife meat products, and indirectly by contact with domestic species following interactions with wildlife species [10]. Outbreak reports of salmonellosis in humans by direct contact with wildlife are numerous and wild reptiles and birds are most commonly implicated [10]. According to the National Notifiable Diseases Surveillance System (NNDSS) of the Australian Government Department of Health [11], a total of 24,267 cases of salmonellosis have been reported in Western Australia (WA) over the last 20 years (2000 to 2020).

Even though the specific sources of contamination are not always identified, macropods and other marsupials may have been the source of infection in at least some of these instances [12]. The quokka (*Setonix brachyurus*) is a small macropod marsupial endemic to Western Australia (WA) [13]. It lives in forested areas in the south-west of mainland WA, but also in high population densities, since European colonisation, on Rottnest Island [14]. This island is within 20 km of the capital city of WA, Perth (population ~2 million), and is a popular tourist destination that attracts ~750,000 visitors a year (2018 data). *Salmonella* was first reported in quokkas from Rottnest Island in 1972 [15], although there was only a low prevalence noted. *S. enterica* ser. Newport was isolated from a single 10-day-old pouch-young.

The first case of human salmonellosis linked to quokkas in WA was recorded in 1973 in a 14-month-old child visiting Rottnest Island; this case was attributed to the consumption of faeces of animal origin and sparked greater interest in the incidence of *Salmonella* on the island [16]. These investigators tested (by enrichment and culture methods) rectal swabs and faecal pellets from quokkas (n = 87) as well as cloacal samples from silver gulls (*Larus novaehollandiae*) (n = 83) from around the site where the child was seen handling faecal pellets, as well as other parts of the island. *S. enterica* ser. Javiana was isolated from the child and the rectal swabs of four quokkas, but not the silver gulls. Of the quokkas sampled, 71% (62/87) were positive to *Salmonella* spp., with 100 isolates (92 isolates of 17 serovars of *S. enterica* subsp. *enterica* and eight isolates of three serovars of *S. enterica* subsp. *arizonae*; Supplementary Table S1).

Subsequently, a 10-year surveillance program was started. This initiative revealed important information about the ecology of *Salmonella* on Rottnest Island [17,18,19,20,21]. Samples were collected from quokkas, reptiles, birds, domestic animals (horses and donkeys), and water sources. A wide diversity of *Salmonella* serovars were detected from 1551 of the 4038 quokkas that were tested (Iveson and Hart, 1983) (Supplementary Table S1). The prevalence of *Salmonella* was lower for birds (10/417; 2.4%), but higher for reptiles (50/76; 66%) and domestic animals (40/45; 88%). The serovars recovered from birds, reptiles, and domestic animals were similar to those isolated from quokkas, indicating that *Salmonella* is both abundant on Rottnest Island and widespread among different animal taxa and ecosystems.

Hart [22] determined that *Salmonella* infections in quokkas had a seasonal variation, with low infection rates (0–30%) in winter and high infection rates (70–100%) during summer. In contrast, non-seasonal infection rates (0–30%) were observed in areas where quokkas had access to supplementary food sources (e.g., near towns). The author suggested that this non-seasonal infection rate may be explained by the ‘urban’ quokka being able to avoid nutritional stress during the driest part of the year. The results of a subsequent experiment supported this assertion [18].

In Australian macropods, salmonellosis typically presents as sepsis or gastroenteritis, and is most commonly noted in captive animals [23]. However, evidence suggests that macropods can also be asymptomatic carriers of *Salmonella* [18,24,25]. Iveson et al. [21] concluded that quokkas can be carriers of *Salmonella*, as recaptured animals remained positive to the same serovar for up to nine months, while Hart et al. [18] and Iveson et al. [17] reported that “the vast majority of infections are believed to be non-disease state” even though no animal was physically examined. Our aim was to correlate *Salmonella* infection with the presence of disease in the Rottnest Island quokka. This was investigated through physical examination, haematology, and blood chemistry profiles. Additionally, we extended our study to mainland WA, where quokkas are further away from urban environments.

## 2. Materials and Methods

### 2.1. Animal Ethics Statement

All animal work was carried out under Murdoch University’s animal ethics permit W2309/10, and Department of Parks and Wildlife (DPaW) Licenses SF007550 and CE002891.

### 2.2. Sample Collection

**Figure 1:**
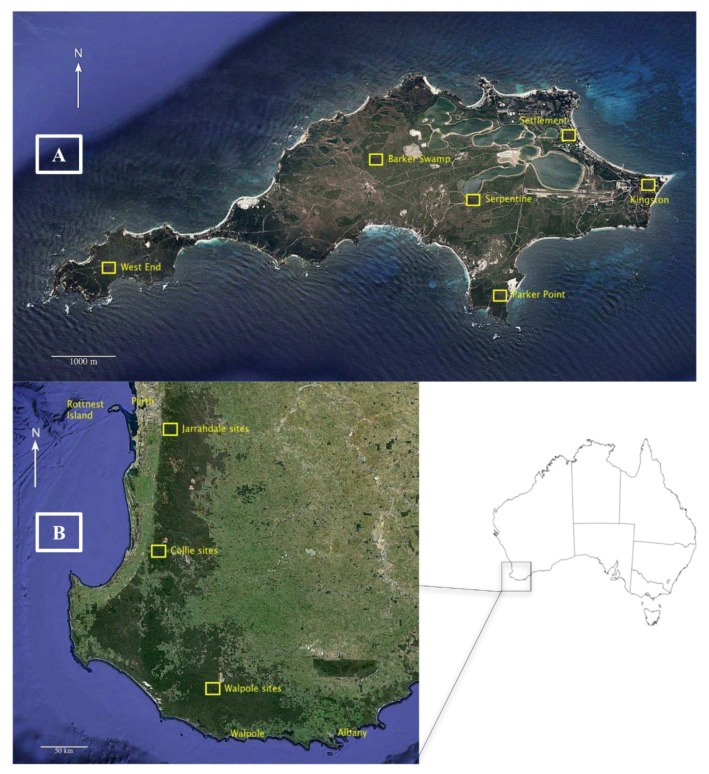
Map of Rottnest Island (**A**) and the mainland (**B**) with the locations where quokkas were trapped. Map data ©2016 Google, Data, Stripss Institute of Oceanography (SIO), National Oceanic and Atmospheric Administration (NOOA), U.S. Navy, National Geospatial Intelligence Agency (NGA), General Bathymetric Chart of the Oceans (GEBCO).

Between September 2010 and December 2011, 92 quokkas were captured and tested for *Salmonella* (71 on Rottnest Island (RI) and 21 on the mainland). Animals were trapped on RI (~1900 ha [26]) with Thomas traps^®^ (Sheffield Wire Products, Welshpool, Australia) from six sites (Figure 1A). Traps were deployed after sunset, baited with peanut butter and oats, and cleared every hour until midnight. Population estimates for RI vary widely, ranging between 8000 and 12,000 individuals [27]. On the mainland, animals were trapped with Sheffield traps^®^ (Sheffield Wire Products) from 11 locations representing three subpopulations, namely Jarrahdale, Collie, and Walpole (Figure 1B), with population estimates of 110, 100, and 700 individuals, respectively [27]. Traps were deployed after sunset, set along water systems, baited with apples, and cleared between 06:00 and 09:00 the following morning.

Animals were removed from traps and transferred to hessian bags for handling. Anaesthesia induction was with 5% isoflurane (I.S.O.^®^, Veterinary Companies of Australia, Kings Park, Australia) delivered in 100% medical oxygen (2.5 L/min). Anesthesia was maintained at 2–3% isoflurane and 2 L/min of oxygen. While anesthetized, samples were collected and a thorough physical examination (PE) was performed on each animal. Following anaesthesia, animals were returned to their natural habitat once mentally alert and ambulatory.

Faecal samples for the isolation of *Salmonella* were obtained by rectal palpation. The external area around the cloaca was cleaned with a 1:1 mixture of chlorhexidine gluconate and 70% ethanol before the procedure. In no cases were faecal samples taken from cotton/hessian bags, or traps. Faecal samples were placed into 5 mL polycarbonate yellow cap sterile tubes (SARSTEDT Aktiengeseilschaft & Co., Nümbrecht, Germany) and stored at 4 °C until processing.

Blood samples from the lateral tail vein were collected in EDTA and lithium heparin blood collection tubes and sent to Murdoch University for haematology using an ADVIA^®^ 120 (Siemens Healthcare Diagnostics, New York, NY, USA) automated analyser and plasma chemistry analyses using an RX Daytona™ automated biochemistry analyser (Randox Laboratories, Kearneysville, WV, USA). Differential counts were done manually on blood smears. Several parameters were recorded and used in this study: red blood cell concentration (RBC); haemoglobin concentration (HGB); packed cell volume (PCV); total white blood cell counts (corrected to exclude nucleated red blood cells) (WBCs); the absolute concentrations for neutrophils, eosinophils, basophils, lymphocytes, and monocytes; and the blood chemistry analytes: alkaline phosphatase (ALP), alanine aminotransferase (ALT), aspartate aminotransferase (AST), creatine kinase (CK), total protein (PROT), albumin (ALB), calcium (CA), phosphorus (P), cholesterol (CHOL), total bilirubin (BILT), glucose (GLUC), creatinine (CREAT), urea (UREA), and vitamin E (Vit. E). Peripheral blood cell (red and white blood cells) morphologies (PBCMs) were visually recorded from blood smears (e.g., reactive lymphocytes, atypical neutrophils (including toxic changes), eccentrocytes, schistocytes), as well as the presence of haemoparasites.

### 2.3. Isolation and Identification of Salmonella spp.

An indirect method with a pre-enrichment step with buffer peptone water (PW), followed by delayed secondary enrichment (DSE) with Rappaport Vassiliadis selective enrichment broth (Oxoid LTD., Hampshire, England), was used for the isolation and identification of *Salmonella* isolates (on the basis of [28]), as well as for the subjective assessment of the bacterial load. Briefly, faecal matter (0.5 g) was placed into 5 mL of buffered PW (i.e., a 1:10 ratio of faeces to buffered PW) and then incubated at 37 °C for 20 h. Vials of RV enrichment broth were then inoculated with 10 µL of PW-faecal mixture and incubated at 42 ± 1 °C for 48 h in a water bath. Fresh Difco™ xylose lysine deoxycholate (XLD) agar plates (BD Diagnostics, Sparks Glencoe, MD, USA) were then inoculated with 30 µL of the PW-RV enrichment broth suspension, streaked out for single colonies, and incubated for 18–24 h at 37 °C. Growth on XLD plates was scored as confluent (no separate colonies), heavy (>200 colonies), moderate (50–200 colonies), or light (<50 colonies or number of colonies). Suspicious colonies were then subcultured onto nutrient agar (NA) plates and incubated for 24 h at 37 °C for further processing.

Suspicious colonies were first confirmed as Gram negative bacilli by Gram’s staining. These isolates were subsequently characterised by a set of biochemical tests (indole (I), methyl red (MR), Voges–Proskauer (VP), citrate (C), urea hydrolysis (U), lactose fermentation (L) and lysine decarboxylase (LD)) to allow discrimination between *Salmonella* spp. and *Shigella* spp. and *Proteus* spp. Lastly, isolates were confirmed as *Salmonella* by an antiserum agglutination test using Antiserum Salmonella Omnivalent Omni-O (A-60) and Antiserum Salmonella Polyvalent OMG (both from Bio-Rad laboratories, Marnes-la-Coquette, France). These two antisera were used to detect agglutination to the presence of somatic (O) antigen of groups O:2 to O:60, and groups O:60 to O:67, respectively. Isolates were stored until further analysis at −80 °C in Protect^®^ Bacterial Preservers cryovials (Technical Service Consultants Limited, Lancashire, United Kingdom).

Following revival by incubation in nutrient broth (NB) at 37 °C for 24 h, NA and XLD agar plates were inoculated and streaked out for single colonies, and incubated at 37 °C for 24–48 h. Isolates were then submitted to the national reference laboratory for *Salmonella* at PathWest (Sir Charles Gairdner Hospital, Western Australia) for serotyping by antisera slide agglutination (Kauffmann–White–LeMinor scheme) to detect O (somatic), H (flagellar), and K (capsular) antigens.

### 2.4. Antimicrobial Susceptibility Testing

Isolates that were confirmed as *Salmonella* and serotyped were submitted to the Animal Health Laboratories (AHL) at the Department of Agriculture and Food of Western Australia (DAFWA). Isolates were tested against amoxicillin (25 µg), amoxicillin-clavulanic acid (30 µg), ceftiofur (30 µg), compound sulphonamides (300 µg), florfenicol (30 µg), lincomycin (2 µg), neomycin (30 µg), penicillin (10 units), tetracycline (30 µg), trimethoprim-sulfamethoxazole (25 µg), tylosin (30 µg), and tulathromycin (30 µg).

### 2.5. Statistical Analyses

Haematology, blood chemistry (BLC), and PBCM datasets for Rottnest Island animals were analysed independently owing to differences in sample sizes between these datasets. Haematology and blood chemistry response variables were fitted to an approximate normal distribution (BoxCox transformation; STATISTICA v. 9.1, StatSoft Inc.). Mainland data were not included as only one animal tested positive to *Salmonella*. Data were explored visually with non-metric multi-dimensional scaling (nMDS) and a Bray–Curtis similarity measure [29] using PAST v. 3.02 [30] comparing between animals that tested positive or negative for *Salmonella*. *Season* and *sex* were used as covariates in all three sets of analyses.

Haematology and BLC dependant variables were range-standardised to a scale between 0 and 1, while PBCMs were not as it was a binary dataset. For each nMDS plot, two-or-three-dimensional analyses were selected according to the model that had the lowest stress statistic to determine adequacy of the fit. To determine the principle differences in HMT, BLC, and PBCM datasets between *Salmonella*-positive and *Salmonella*-negative quokkas, a similarity percentage (SIMPER, PAST v. 3.02) analysis [31] was carried out using a Bray–Curtis similarity measure [29]. SIMPER results (i.e., percent of contribution of each variable to the similarity or dissimilarity) are accompanied by the arithmetic mean ($\bar{x}$) and standard deviation (SD) for each HMT and BLC variable, while odds ratio (OR) and 95% confidence intervals (CIs) were calculated [32] for each PBCM observed in blood smears. To determine the significance of any differences between dependant variable communities (HMT, BLC, and PBCM) as a function of *Salmonella*, a two-way non-parametric permutational multivariate analysis of variance (two-way PERMANOVA, PAST v. 3.02) [33] with 9999 permutations was subsequently run. The effect size (ES) *Cohen’s d* was calculated using the *F*-value given by PERMANOVA or the *χ*^2^ value given by Chi-square analysis [34]. Clinically, the magnitude of the ES was considered to be small if *d* ≤ 0.2, moderate if 0.2 > *d* < 0.8, and large if *d* ≥ 0.8 [35].

Seasonal differences in the prevalence of *Salmonella* infections on Rottnest Island were explored using *χ*^2^ for trend. Associations between *Salmonella* and *sex* (across Rottnest Island) were explored using *χ*^2^ with Yates’ correction. Odd ratios and 95% CIs were calculated using the Woolf’s method [32]. When a null value was present in a contingency table, 0.5 was added to each observed value in order to calculate OR and 95% CIs [36]. Additionally, ES (*Cohen’s d*) was calculated when considered appropriate. All other CIs for estimates of proportions (i.e., prevalence) were calculated using the Wilson model for n ≤ 40, and the Jeffreys model for n > 40 [37]. Significance was set at *p* < 0.05 for all analyses, unless stated otherwise.

## 3. Results

### 3.1. Detection of Salmonella spp.

The morphologies of all *Salmonella* serovars isolated in this study were considered to be typical for XLD agar: pink to red with black centres [38] (Supplementary Figure S1). Colonies were mostly circular, effuse, smooth, and translucent. They were Gram-negative bacilli on Gram’s stain and presented a typical biochemical profile for *Salmonella*. All isolates exhibited positive agglutination (Supplementary Figure S1) to antiserum Salmonella Omnivalent Omni-O (A-60) and no agglutination to antiserum Salmonella Polyvalent OMG, indicating isolates were not serovars of the somatic (O) antigen groups 61 to 67.

Of the 35 animals that were positive to *Salmonella* (Rottnest Island = 34, mainland = 1), 28 (80%) had a confluent growth on XLD (RV selective enrichment inoculum) at 24 h post incubation, while there was a heavy growth of the isolates from the remaining seven animals (i.e., >200 colonies).

Of the 21 individuals (18 male and 3 female) from mainland sites, an adult male was positive to *S. enterica* subsp. *diarizonae* (IIIb) ser. 50:k:z_35_. Of the 71 individuals on Rottnest Island tested (40 male and 31 female), 34 animals were positive to *Salmonella* (prevalence 47.9%). Of these 34 animals, 18 were males (prevalence n = 40 = 45%, CI 36.7–68.5) and 16 were females (prevalence n = 31 = 51%, CI 31.5-63.3). The probability (i.e., OR) of females being positive to *Salmonella* was 1.30 (CI 0.51–3.34) times higher than males. However, this association was not significant (*χ*^2^_1, n=71_= 0.30, *p* = 0.581).

**Figure 2:**
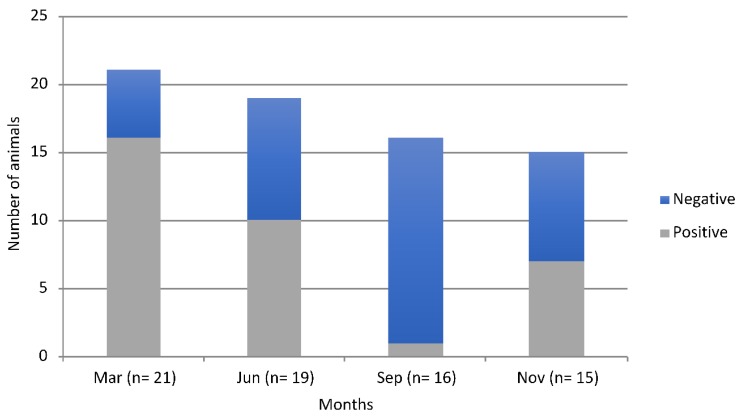
Monthly proportion of *Salmonella* spp. positive and negative animals on Rottnest Island between March and December of 2011.

There was seasonal variation (*χ*^2^_1, n=71_= 7.65, *p* = 0.001) in the prevalence of *Salmonella* in Rottnest Island animals (Figure 2) with the highest prevalence of infection at the end of the dry summer season (March), while the lowest prevalence was at the end of the wet winter (September).

A total of 37 isolates were obtained from 35 animals (Table 1). Only one of these isolates was detected from mainland quokkas. Among the 37 isolates, 16 serovars were identified. Of these, 13 serovars (81.25%, CI 57–93.4) belonged to *S. enterica* subsp. *enterica*, one serovar (6.25%, CI 1.1–28) to *S. enterica* subsp. *salamae* (II), and two serovars (12.5%, CI 3.2–36) to *S. enterica* subsp. *diarizonae* (IIIb). Two serovars were recovered simultaneously from two Rottnest Island animals: *S. enterica* ser. Adelaide and *S. enterica* ser. Cerro were detected in one animal, and *S. enterica* ser. Chester with *S. enterica* ser. Cerro were detected in the other animal. Of the 36 isolates obtained from Rottnest Island, *S. enterica* ser. Adelaide (19.4%, CI 10–35) and *S. enterica* ser. Muenchen (13.9%, CI 6.1–29) were the most frequently detected serovars. This was followed by *S. enterica* ser. Cerro (11.1%, CI 3.9–25). A total of five new *Salmonella* serovars were isolated from quokkas across Rottnest Island and mainland samples.

### 3.2. Antibiotic Susceptibility Test Results

All isolates of each serovar were tested against a panel of antibiotics. Serovars of all three *S. enterica* subspecies isolated (*enterica*, *salamae* and *diarizonae*) were found to have a similar antimicrobial susceptibility profile. The serovars were susceptible to amoxicillin, amoxicillin-clavulanic acid, potentiated and non-potentiated sulphonamides, ceftiofur, florfenicol, neomycin, tetracycline, and tulathromycin. All serovars were resistant to lincomycin, penicillin, and tylosin.

### 3.3. Associations of Salmonella with Haematology, Blood Chemistry, and PBCM Variables for Rottnest Island Quokkas

Blood chemistry was significantly different between *Salmonella*-positive and *Salmonella*-negative animals (PERMANOVA: *p* = 0.05, Table 2 and Table 3) with higher ALP, BILT, CREAT, and UREA, and lower GLUC, PROT, and ALB in *Salmonella*-positive animals. There was weak evidence for differences in haematology (WBC (lower LYMPH, EOS, and NEUT; and higher MONO), erythrogram (lower RBC, HGB, and PCV)) between *Salmonella*-positive and *Salmonella*-negative animals (PERMANOVA: *p* = 0.07, Table 2 and Table 3). There was no difference between *Salmonella*-positive and *Salmonella*-negative animals for peripheral blood cell morphology (Table 2 and Table 3).

There was no particular clustering of the variables (HMT, BLC, and PBCM) either across seasons or across sexes (nMDS analyses; Supplementary Figure S2). In contrast, there was a consistent and significant effect (PERMANOVA: *p* < 0.01; Table 3) of season and sex on BLC profiles, whereas season was the only factor to have a significant effect (*p* < 0.01) on PBCM of quokkas on Rottnest Island. No clustering was evident in their respective nMDS plots (Supplementary Figure S2).

### 3.4. Associations of Salmonella with Physical Examination Findings

There were no differences in the physical examination findings between animals positive or negative for *Salmonella* (Table 4).

## 4. Discussion

This report describes the isolation of *Salmonella* from free-ranging quokkas and, for the first time, their association with the health of the quokka. Sixteen serovars of *Salmonella* were recovered in the present study. *Salmonella enterica* ser. Adelaide and ser. Muenchen were detected most often, followed by ser. Cerro. Of all the serovars recovered, while five had not been reported previously in quokkas. In total, *Salmonella* spp. were detected in 47.9% (34/71) of quokkas from Rottnest Island, but only in 4.8% (1/21) of mainland quokkas that were tested.

It is known that animals infected with *Salmonella* can intermittently shed the bacterium in their faeces [39,40]. We observed a seasonal pattern of *Salmonella* infections on Rottnest Island. The lowest and highest prevalences were observed in winter and summer, respectively. Similar observations have been documented previously in the Rottnest Island quokka [16,17,21]. Increased *Salmonella* shedding by infected animals [41], as well as increased serovar diversity [42], have been linked with the dry conditions seen in summer. Our findings also indicated that the previously reported association between settled areas and a low prevalence of *Salmonella* in quokkas [17,18,21] may still persist. The different rates of infection seen between settled and unsettled areas were attributed by the authors to the year-round supplementary feeding that animals near to settlements receive [18,22]. This is assumed to be from access to the rubbish disposal site and from visitors. Consistent with these findings, we too observed a lower prevalence of infection in heavily human-intervened sites (i.e., Settlement: 30.77%, n = 13), while a higher prevalence was observed in less human-intervened sites (i.e., Serpentine: 66.66%, n = 12, Barker Swamp: 63.63%, n = 11).

The prevalence of *Salmonella* obtained in this study for mainland animals (4.8%) is considerably lower than that previously reported (63.6%) [22]. It is important to note though that this previous study screened faecal samples that were collected from the ground, and so linking the isolation of *Salmonella* to the prevalence of *Salmonella* in quokkas (as opposed to the prevalence in faecal samples) becomes problematic. Additionally, some animals were manipulated with equipment and holding bags that had previously been used on Rottnest Island [22], where the two serovars most commonly detected in their study (*S. enterica* ser. Orientalis and ser. Newbrunswick) appear to be among the most prevalent [17]. In our study, we aimed to obtain reliable results through a number of practices. First, faecal pellets were collected directly from the animal by rectal palpation after disinfecting the pericloacal region. Second, all handling equipment used on mainland animals was exclusive for mainland fieldwork. Third, all medical equipment (e.g., anaesthetic equipment) was chemically sterilised with F10SCXD (Health and Hygiene Pty. Ltd., Florida Hills, South Africa) between trapping sessions. A similar prevalence to what we identified in mainland quokkas (4.2%) was seen in Western Australian western grey kangaroos (*Macropus fuliginosus*) (3.6%) [24]. Despite the differences identified between our study and the study by Hart [22], we are unable to provide a compelling explanation for why our respective prevalence data differ so greatly. It should be noted though that the number of mainland quokkas in our study was low, as this population is very elusive and difficult to trap. A larger sample of mainland quokkas, collected over multiple seasons and years, would provide a more accurate estimate of the true prevalence in the mainland population and how it varies over time.

For the Rottnest Island population of quokkas in our study, the widespread detection of *Salmonella* was consistent with previous studies [16,17,21].

There was weak evidence (*p* = 0.05–0.08) for associations between the detection of *Salmonella* and the haematology and blood chemistry response variables. However, there was no association between physical examination findings and the detection of *Salmonella* spp. as, generally, all animals in this study were apparently healthy, with no obvious signs of clinical salmonellosis. With the exception of PCV, RBC, and HGB, which may be attributable to the seasonal anaemia experienced by quokkas on Rottnest Island [43,44], and the increased CALC concentrations in serum, the overall trend observed in all other parameters (i.e., haematology and blood chemistry) between *Salmonella*-positive and *Salmonella*-negative quokkas resembles the changes seen in bovine calves with clinical salmonellosis owing to *S. enterica* ser. Typhimurium [45]. Given the absence of physical examination findings consistent with *clinical* salmonellosis, it is possible that the presence of *Salmonella* in quokkas in this study can be associated with a *subclinical* salmonellosis.

Rectal swabs have been used instead of faecal pellets by other investigators, but we analysed faecal pellets instead as this sample type has been shown to produce better results for isolating low numbers of *Salmonella* [18]. However, it does have the obvious disadvantage that an animal can only be sampled if faeces are found in the rectum. In our study, this precluded 61 other animals from being screened for *Salmonella*. Future studies could utilise both sample types, but this would need to be balanced against the disadvantage of adding an additional variable into the experiment (i.e., sample type).

We did not screen samples using PCR-based testing. It is difficult to predict what impact this would have had on our results if we had included this testing modality. There is not a consensus on whether PCR reliably outperforms culturing *Salmonella*; some studies have favoured PCR [46,47], others have favoured culture [48], and some could not conclude that one method was superior to the other [49,50]. Although quicker results can be obtained through PCR-based methods [48,49,51], and in some cases with a greater degree of sensitivity, for epidemiology studies, PCR methods have not, and cannot, reliably replace culture methods and serotyping [52,53,54], given that less than 10% of today’s known serovars can be identified to the serovar level by PCR methods.

A weakness of this study was that only Rappaport–Vassiliadis (RV) selective enrichment broth was used for the delayed secondary enrichment culturing step. This is inappropriate for the detection of non-motile *Salmonella* [55]. Though non-motile strains of *Salmonella*, which are generally mutants (with the exception of *S. enterica* subsp. *enterica* ser. Gallinarum) that either synthesise a non-motile flagellum or do not synthesise a flagellum at all, may not have been isolated, these mutant forms are likely to be of no or little concern for the health of quokkas, given that the absence of motility compromises bacterial colonisation [56,57,58]. Despite this, future studies should consider using methods of *Salmonella* culture that have recently been described [55]. These robust methods have specificity, sensitivity, and detection limits that are comparable between laboratories [59].

Previous studies have suggested female quokkas may be more susceptible to infection with *Salmonella* than males [21,22]. The prevalence of *Salmonella* in quokkas on Rottnest Island peaks between November and March (based on our data), which coincides with the female’s gestation, births, and lactation events [60]. In our study, there was no evidence to support that females were more susceptible to *Salmonella* than males (*p* = 0.58), although we tested very few females from mainland populations.

## 5. Conclusions

From a conservation perspective, it is difficult to predict whether or not *Salmonella* infections could become a threatening process to the Rottnest Island quokka. Habitat clearing and increased visitation to the island could provide stressors to the endemic quokka population, which in turn could precipitate disease associated with the *Salmonella* spp. many of them carry. From a public health point of view, tracking the prevalence and incidence of *S. enterica* subsp. *enterica* serovars on Rottnest Island is recommended to facilitate public health management. Our study may thus serve as a template for future studies that enable long-term monitoring of *Salmonella* in quokkas.

## Figures and Tables

**Table 1:** Distribution and number of *Salmonella* isolations from quokkas on Rottnest Island and the mainland between June 2010 and December 2011.

Serovar Isolated	Barker Swamp	Kingston	Parker Point	Serpentine	Settlement	West End	Mainland	Totals
*S. enterica* subsp. *enterica*								
ser. Adelaide			2	3		2		7
ser. Alachua †				1				1
ser. Bootle						1		1
ser. Bredeney †	1			1				2
ser. Carnac					2			2
ser. Cerro †		1	1	1	1			4
ser. Chester	1		1	1				3
ser. Choleraesuis var. Decatur				1				1
ser. Infantis		1			1			2
ser. Muenchen	1	2				2		5
ser. Orion	1			1				2
ser. Rottnest		1						1
ser. Waycross	1							1
*S. enterica* subsp. *diarizonae*								
(IIIb) ser. 35:k:z_53_ †	1							1
(IIIb) ser. 50:k:z_35_ ‡							1	1
*S. enterica* subsp. *salamae*								
(II) ser. 21:z_10_:z_6_	1	1	1					3
*Total no. of animals tested*	11	12	11	12	13	12	21	92
*Animals positive*	*7*	*6*	*4*	*8*	*4*	*5*	*1*	*35*
*No. of isolations*	*7*	*6*	*5*	*9*	*4*	*5*	*1*	*37*

† new *Salmonella* serovars isolated from quokkas on Rottnest Island, ‡ new *Salmonella* serovar isolated from quokkas on the mainland.

**Table 2:** SIMPER analysis results indicating the contribution of specific variables to the observed differences in HMT (a), BLC (b), and PBCM (c) profiles of quokkas that were *Salmonella*-positive and *Salmonella*-negative on Rottnest Island. HMT = haematology, BLC = blood chemistry, PBCM = peripheral blood cell morphology, RBC = red blood cell concentration, WBC = white blood cell count, HGB = haemoglobin concentration, PVC = packed cell volume, ALP = alkaline phosphatase, ALT = alanine aminotransferase, AST = aspartate aminotransferase, CK = creatine kinase, PROT = total protein, ALB = albumin, CA = calcium, P = phosphorus, CHOL = cholesterol, BILT = total bilirubin, GLUC = glucose, CREAT = creatinine, UREA = urea, Vit. E = vitamin E.

		Taxon	Ct %	*Salmonella* +ve		*Salmonella* -ve	
				$\bar{x}$	SD	$\bar{x}$	SD
a.	HMT (31) † n +ve = 26 n −ve = 31	Lymphocytes (×10^9^/L)	13.52	1.66	0.79	2.03	1.03
		RBC (×10^12^/L)	11.98	5.60	0.92	5.85	0.78
		WBC (×10^9^/L)	11.84	4.20	1.55	4.94	1.60
		Monocytes (×10^9^/L)	11.76	0.07	0.04	0.06	0.06
		Basophils (×10^9^/L)	11.72	0.02	0.02	0.02	0.02
		Eosinophils (×10^9^/L)	11.01	0.32	0.28	0.51	0.49
		HGB (g/L)	9.978	105	16.4	108	12.3
		Neutrophils (×10^9^/L)	9.652	2.13	0.97	2.32	1.12
		PCV (%)	8.544	32.7	4.43	33.5	4.79
b.	BLC (20.9) † n +ve = 30 n −ve = 36	Vit. E (mg/L)	9.55	6.89	1.74	6.44	1.63
		CK (U/L)	9.12	861	768	949	1035
		PHOS (mmol/L)	8.83	1.01	0.34	1.2	0.46
		ALP (U/L)	7.96	11,278	15,617	5433	3308
		CHOL (mmol/L)	7.75	2.56	0.47	2.88	0.52
		GLUC (mmol/L)	7.40	4.20	2.01	4.27	2.71
		PROT (g/L)	7.14	60.2	3.67	60.8	5.59
		BILT (µmol/L)	6.91	5.01	2.05	4.39	1.47
		ALT (U/L)	6.87	222	76.7	222	62.4
		CALC (mmol/L)	6.77	2.24	0.18	2.19	0.23
		ALB (g/L)	6.49	36.1	1.44	36.3	1.97
		AST (U/L)	5.36	54.1	23.6	45.7	17.6
		CREAT (µmol/L)	5.12	74.1	17.9	69.4	15
		UREA (mmol/L)	4.75	7.10	1.87	6.88	1.67
				*Salmonella* +ve	*Salmonella* −ve		
			Ct %	Frequency (%)	Frequency (%)	OR ‡ (95% CI)	
c.	PBCM (24.59) † +ve = 30 −ve = 36	Rouleaux formation	12.2	16 (53.3)	16 (44.4)	1.43 (0.54–3.78)	
		Acanthocytes	12.1	17 (56.7)	19 (52.8)	1.17 (0.44–3.10)	
		Heinz bodies	10.8	11 (36.7)	12 (33.3)	1.16 (0.42–3.20)	
		Schistocytes	8.57	8 (26.7)	8 (22.2)	1.27 (0.41–3.93)	
		Anisocytosis	8.18	26 (86.7)	27 (75)	2.17 (0.59–7.91)	
		Keratocytes	8.12	7 (23.3)	8 (22.2)	1.07 (0.34–3.38)	
		Flower Cells	8.01	4 (13.3)	10 (27.8)	0.40 (0.11–1.44)	
		Echinocytes	7.80	6 (20)	8 (22.2)	0.88 (0.27–2.88)	
		Poikilocytosis	6.61	25 (83.3)	31 (86.1)	0.81 (0.21–3.10)	
		Hypochromasia	5.18	27 (90)	31 (86.1)	1.45 (0.32–6.65)	
		nRBCs	4.84	27 (90)	32 (88.9)	1.13 (0.23–5.47)	
		Polychromasia	3.87	28 (93.3)	33 (91.7)	1.27 (0.20–8.17)	
		Howell-Jolly bodies	2.84	29 (96.7)	33 (91.7)	2.64 (0.26–26.7)	
		Reactive lymphocytes	0.90	30 (100)	35 (97.2)	2.58 (0.10–65.6) *	

Ct: percent of contribution to difference, † overall average dissimilarity, ‡ odds ratio (OR) for the presence of the taxon in *Salmonella* +ve individuals and 95% confidence intervals (CIs), calculated using Woolf’s method [32], * calculated by adding 0.5 to each observed value [36].

**Table 3:** Two-way permutational multivariate analysis of variance (PERMANOVA) of selected HMT variables (a) (corrected WBC, RBC, HGB, PCV, and absolute counts for leukocytes obtained with a manual differential on a blood smear), BLC analytes (b) (ALP, ALT, AST, CK, PROT, ALB, CALC, PHOSP, CHOL, BILT, GLUC, CREAT, UREA, and vitamin E), and PBCM (c), for quokkas that were positive and negative to any *Salmonella* serovar, with season and sex as independent factors. Bray–Curtis similarity index, permutations N = 9999. Only two independent factors could be tested simultaneously, and thus the presence of *Salmonella* was tested first with season, and then secondly with sex of the animal. HMT = haematology, BLC = blood chemistry, PBCM = peripheral blood cell morphology.

		Data	Factor	SS	df	MS	*F*	*p*
a.	All *Salmonella* serovars	HMT	Factor *Salmonella*	0.108	1	0.108	1.31	0.08 †
			Factor Season	0.179	3	0.060	0.724	0.39
			Interaction	−1.24	3	−0.414	−5.03	0.82
			Residual	4.03	49	0.082		
			Total	3.08	56			
			Factor *Salmonella*	0.108	1	0.108	1.90	0.07 †
			Factor Sex	0.061	1	0.061	1.08	0.34
			Interaction	−0.111	1	−0.111	−1.95	0.45
			Residual	3.02	53	0.057		
			Total	3.08	56			
b.		BLC	Factor *Salmonella*	0.039	1	0.039	1.46	**0.05** ‡
			Factor Season	0.215	3	0.072	2.70	**0.01**
			Interaction	−0.320	3	−0.107	−4.02	0.25
			Residual	1.54	58	0.027		
			Total	1.47	65			
			Factor *Salmonella*	0.039	1	0.039	1.69	0.07 ‡
			Factor Sex	0.063	1	0.063	2.75	**0.01**
			Interaction	−0.05	1	−0.05	−2.20	0.34
			Residual	1.42	62	0.023		
			Total	1.47	65			
c.		PBCM	Factor *Salmonella*	0.046	1	0.046	0.898	0.36
			Factor Season	0.409	3	0.136	2.678	**0.01**
			Interaction	−0.752	3	−0.251	−4.93	0.95
			Residual	2.95	58	0.051		
			Total	2.65	65			
			Factor *Salmonella*	0.015	1	0.015	0.340	0.80
			Factor Sex	0.089	1	0.089	1.98	0.09
			Interaction	−0.252	1	−0.252	−5.60	0.99
			Residual	2.80	62	0.045		
			Total	2.65	65			

SS: sum of squares, Df: degrees of freedom, MS: mean sum of squares, † effect size for haematology *Cohen’s d* = 0.34 (moderate), ‡ effect size for blood chemistry *Cohen’s d* = 0.32 (moderate).

**Table 4:** Associations between physical examination findings and the presence of *Salmonella* in animals from Rottnest Island. OR = odds ratio, CI = confidence interval, CRT = capillary refill time, MM = mucous membranes, EP = external parasites.

Finding	*Salmonella* spp.		OR † (95% CI)	*χ²*_1, n = 71_
	+ve (%) _n = 34_	−ve (%) _n = 37_		
Abnormal mentation ^a^	1 (1.41)	0 (0)	3.36 (0.13–85.3) *	= 1.10, *p* = 0.293
Abnormal CRT ^b^	4 (5.63)	2 (2.81)	2.33 (0.40–13.6)	= 0.92, *p* = 0.335
Abnormal MM ^c^	10 (14.08)	10 (14.08)	1.13 (0.40–3.17)	= 0.05, *p* = 0.823
Cloudy eye	1 (1.41)	1 (1.41)	1.09 (0.07–18.1)	= 0.43, *p* = 0.511
Dehydration ^d^	16 (22.53)	17 (23.94)	1.05 (0.41–2.66)	= 0.01, *p* = 0.925
Ear notches	5 (7.04)	10 (14.14)	0.47 (0.14–1.54)	= 1.61, *p* = 0.203
Flaky skin	1 (1.41)	0 (0)	3.36 (0.13–85.3) *	= 0.01, *p* = 0.966
Fur loss	11 (15.49)	6 (8.45)	2.47 (0.80–7.66)	= 2.53, *p* = 0.111
Presence of EP	9 (12.67)	5 (7.04)	2.30 (0.69–7.74)	= 1.88, *p* = 0.170
Skin erosions	2 (2.82)	1 (1.41)	2.25 (0.19–26.0)	= 0.01, *p* = 0.940
Wheezes	0 (0)	1 (1.41)	0.35 (0.01–8.95) *	= 0.01, *p* = 0.966

^a^ Diminished response to external stimuli, ^b^ greater than two seconds, ^c^ pale and blue tinted mucous membranes, ^d^ skin tenting for longer than 2 s, † odds ratio for the presence of the taxon in *Salmonella* +ve individuals and 95% confidence intervals, calculated using Woolf’s method [32], * calculated by adding 0.5 to each observed value [36].

## Supplementary Materials

The following are available online at https://www.mdpi.com/2076-2615/10/4/585/s1, Figure S1: Growth on XLD agar plates and antiserum agglutination test., Table S1: *Salmonella* serovars isolated from quokkas (*Setonix brachyurus*).
