# Supplementary material for: Salmonella in Free-Ranging Quokkas (Setonix brachyurus) from Rottnest Island and the Mainland of Western Australia
Source: Animals (Basel). 2020 Mar 31;10(4):585. doi: 10.3390/ani10040585 (PMC7222713; doi:10.3390/ani10040585)
Supplement: Supplementary file 1 [file animals-10-00585-s001.pdf]

Table S1. *Salmonella* [1] serovars isolated from quokkas (*Setonix brachyurus*) from faecal pellets (F) (collected from the animal A, post-mortem PM, ground G, or undetermined U), Rectal swabs (R), Intestinal (I), and Undetermined (U) samples from Rottnest Island (RI), Bald Island (BI) or the mainland (M).

Serovar		Yadav et al. [15] [2]	Iveson and Bradshaw [16]	Hart et al. [61]	Iveson and Hart [17]	Hart et al. [18]	Hart et al. [19]	Iveson et al. [21] [3]	Martínez-Pérez [62]
Nomenclature									
Original paper	White-Kauffmann-Le Minor	RI	RI	RI	RI	RI	M & BI	RI	RI & M
S. adelaide	*S. enterica* ser. Adelaide		R	R F^{U}	U	G F^{PM}		R	F^{A}
S. alsterdorf or *II alsterdorf* [4]	II 1,40:g:[m],[s],t:[1,5]			R F^{U}	U			R	
	S. enterica ser. Alachua								F^{A}
S. anatum	*S. enterica* ser. Anatum		G		U			R	
S. bahrenfeld	*S. enterica* ser. Bahrenfeld		R	R F^{U}	U			R	
S. birkenhead	*S. enterica* ser. Birkenhead							R	
S. bleadon	II 17:g,t:[e,n,x,z_{15}]			R F^{U}	U				
S. blukwa	*S. enterica* ser. Blukwa			R F^{U}	U				
S. bootle	*S. enterica* ser. Bootle		R		U			R	F^{A}
S. bovis-morbificans	*S. enterica* ser. Bovismorbificans			R F^{U}	U		R (BI)	R	
	S. enterica ser. Bredeney								F^{A}
S. bunnik	II 43:z_{42}:1,5,7			R F^{U}	U				
S. carnac	*S. enterica* ser. Carnac						R G (BI)		F^{A}
S. charity	*S. enterica* ser. Charity						R G (BI)		
	S. enterica ser. Cerro								F^{A}
S. chester	*S. enterica* ser. Chester		R G	R F^{U}	U			R	F^{A}
S. decatur	*S. enterica* ser. Choleraesuis var. Decatur		G	R F^{U}	U			R	F^{A}
S. derby	*S. enterica* ser. Derby			R F^{U}	U				
S. fremantle	II 42:g,t:-		R		U				
S. give	*S. enterica* ser. Give		R	R F^{U}	U				
S. havana	*S. enterica* ser. Havana		R G	R F^{U}					
S. infantis	*S. enterica* ser. Infantis			R F^{U}	U			R	F^{A}
S. javiana	*S. enterica* ser. Javiana		R G	R F^{U}	U	F^{PM}		R	
S. merseyside	II 16:g,t:[1,5]						G (BI)		
S. muenchen	*S. enterica* ser. Muenchen		R G	R F^{U}	U	R F^{PM}		R	F^{A}
S. newbrunswick	*S. enterica* ser. Give var. 15^{+}		R				G (M)		

S. newington	*S. enterica* ser. Newington		R G	R F^{U}		R F^{PM}		R	
S. newport	*S. enterica* ser. Newport	I^{PM}		R F^{U}	U				
S. oranienburg	*S. enterica* ser. Oranienburg		R	R F^{U}	U	R		R	
S. orientalis	*S. enterica* ser. Orientalis		R	R F^{U}	U	F^{PM}	G (M)	R	
S. orion	*S. enterica* ser. Orion		R	R F^{U}	U			R	F^{A}
S. potsdam	*S. enterica* ser. Potsdam			R F^{U}	U			R	
S. rottnest	*S. enterica* ser. Rottnest			R F^{U}	U			R	F^{A}
S. saint-paul or saintpaul	*S. enterica* ser. Saintpaul			R F^{U}	U				
S. singapore	*S. enterica* ser. Singapore			R F^{U}	U	F^{PM}			
S. typhimurium	*S. enterica* ser. Typhimurium		R G	R F^{U}	U	R	G(BI)	R	
S. wandsbek or *II wandsbek*[5]	II 21:z_{10}:z_{6}		R	R F^{U}	U	R F^{PM}		R	F^{A}
S. waycross or *IV waycross*[6]	*S. enterica* ser. Waycross		R	R F^{U}	U	R F^{PM}		R	F^{A}
S. 6,8:-:- (O Group C_{2})	-						R (BI)		
S. 48:d:-	-			R F^{U}	U			R	
53:d:z_{42}	II 53:d:z_{42}							R	
Arizona spp.					U				
IIIb 25:l v:z_{53}	-							R	
IIIb 50:K:z_{35}	IIIb 50:K:z_{35} [7]							R	F^{A} (M)
IIIb 61:l v:z_{35}	IIIb 61:l,v:z_{35}							R	
IIIb 61:z_{52}:z_{53}	IIIb 61:z_{52}:z_{53}							R	
A. 9:26:21	IIIb 50:z_{52}:z_{35} [7]				U				
A. 9:26:31	-				U				
A. 9:29:21	-				U				
A. 9a9b:26-21	IIIb 50:z_{52}:z_{35} [7]			R F^{U}					
A. 9a9b:26-31	IIIb 50:z_{52}:z [8]			R F^{U}					
A. 9a9b:29-21	IIIb 50:K:z_{35} [7]			R F^{U}					
A. 16:23:25	IIIb 38:lv:z53 [9]		R		U				
A. 16:26:25	IIIb 38:z52:z53 [9]				U				
A. 20:22-21	IIIb 35:(k):z35 [9]			R F^{U}					
A. 20:29:21	-				U				
A. 20:29:25	IIIb 35:(k):z_{53}:[z_{50}] [10]			R F^{U}	U				F^{A}
A. 26:23:21	IIIb 61:l,v:z_{35} [7]		R	R F^{U}	U				
A. 26:23:25	-		R						
A. 26:26-25	IIIb 61:z52:z53 [9]			R F^{U}					
A. 28:32:28	IIIb 47:c:e,n,x,z15 [8]			R F^{U}	U				

[1] Serovars previously classified as genus *Arizona* (A.) are included in this table because they have since been reclassified into the genus *Salmonella*, [2] *Salmonella* was isolated from a 10-day old pouch young at post-mortem, [3] In this study, samples were only collected from *S. brachyurus* that had been captured at sites considered to have high human-animal interaction rates i.e. settlements, camping areas etc, [4] This serovar was referred to as *S. alsterdorf i*n Iveson and Bradshaw [16] and *II alsterdorf* in Iveson et al. [21], [5] This serovar was referred to as *S. wandsbek* in Iveson and Bradshaw [16] and *II Wandsbek* in Iveson et al. [21], [6] This serovar was referred to as *S. waycross* in Iveson and Bradshaw [16] and *IV Waycross* in Iveson et al. [21], [7] Current *Salmonella* antigenic formula was obtained from Hall and Rowe [63], [8] Patricia Fields, CDC Atlanta *pers. comm*. 2013, [9] Damien Bradford, PathWest, Perth *pers. comm.* 2013, [10] Current *Salmonella* antigenic formula was obtained from Weis et al. [64].

Reference

61. Hart, R.P.; Iveson, J.B.; Bradshaw, S.D.; Speed, T.P. A study of isolation procedures for multiple infections of *Salmonella* and *Arizona* in a wild marsupial, the quokka (*Setonix brachyurus*). *J. Appl. Bacteriol.* **1982**, *53*, 395–406.
62. Martínez-Pérez, P. Health and disease status in a threatened marsupial, the quokka (*Setonix brachyurus*). Murdoch University, 2016.
63. Hall, M.L.M.; Rowe, B. *Salmonella* arizonae in the United Kingdom from 1966 to 1990. *Epidemiol. Infect.* **1992**, *108*, 59–65.
64. Weiss, S.H.; Blaser, M.J.; Paleologo, F.P.; Black, R.E.; McWhorter, A.C.; Asbury, M.A.; Carter, G.P.; Feldman, R.A.; Brenner, D.J. Occurrence and distribution of serotypes of the Arizona subgroup of *Salmonella* strains in the United States from 1967 to 1976. *J. Clin. Microbiol.* **1986**, *23*, 1056–1064.

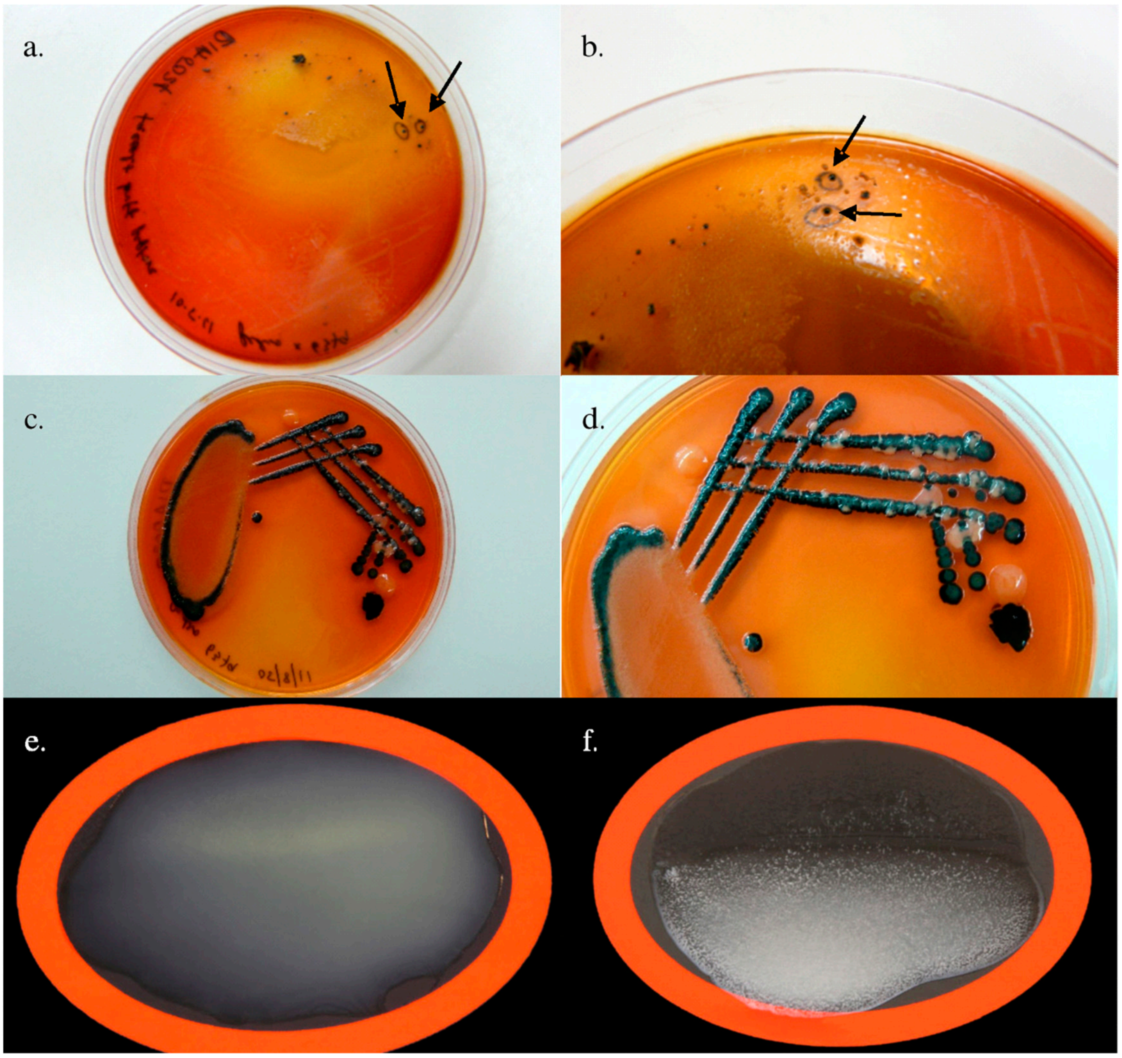

Figure S1. Growth on XLD agar plates and antiserum agglutination test. (**a**,**b**) suspicious *Salmonella* colonies on an XLD agar plate inoculated with a suspension of PW (pre-enrichment) and faeces. Colonies are indicated by closed arrows. (**c**,**d**) suspicious *Salmonella* colonies on an XLD agar plate inoculated with a suspension of pre-enrichment and Rappaport Vassiliadis selective enrichment broth, and streaked out for single colonies. Note colonies of other intestinal bacteria and the predominant and suspicious colonies with H_2S precipitate. (**e**) negative agglutination test for *Salmonella*, (**f**) positive agglutination test for *Salmonella* with obvious clumps in the homogenate.

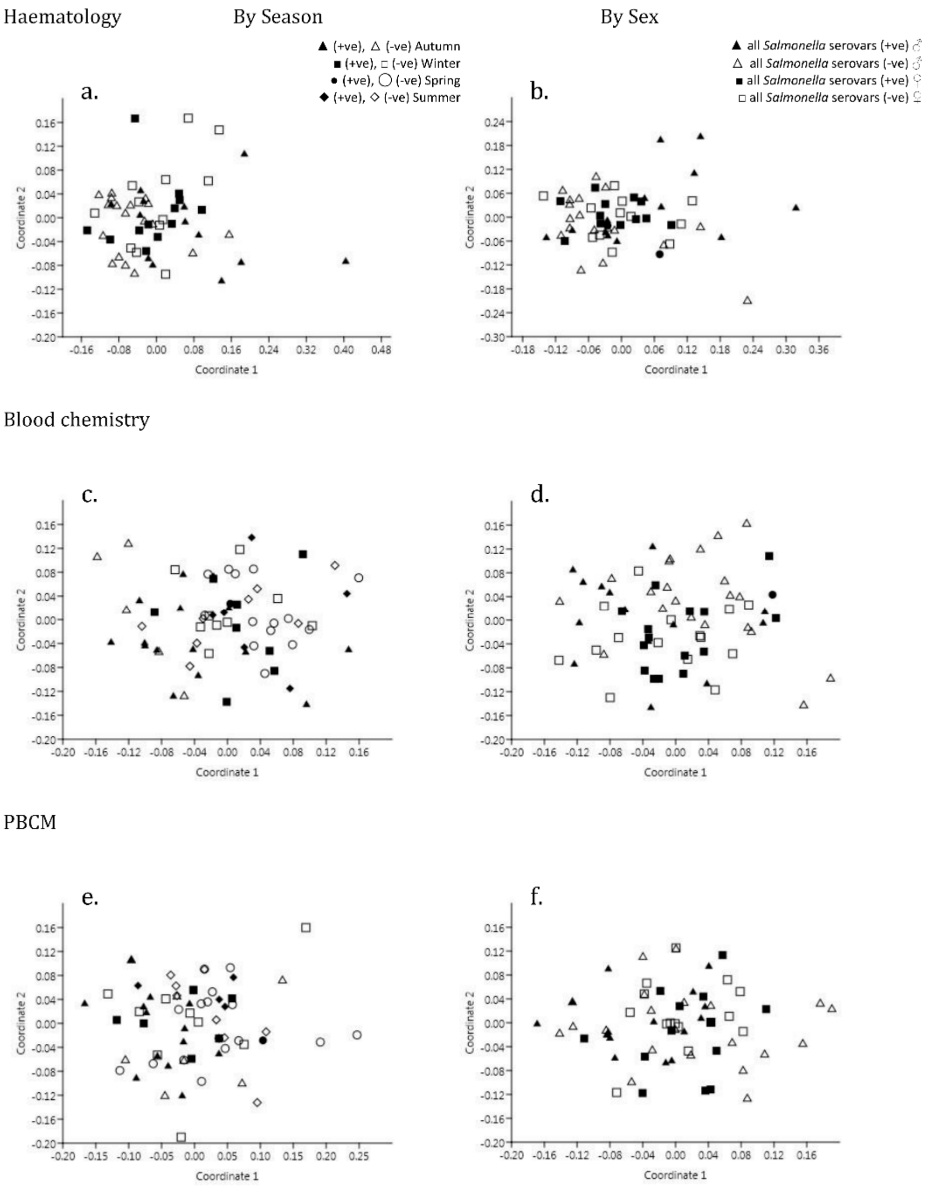

Figure S2. Non-metric MDS plots with Bray-Curtis similarity measure, illustrating the structural dissimilarity of haematology (a, b: stress statistic = 0.158), blood chemistry (c. stress statistic = 0.273; d. stress statistic = 0.238), and PBCM communities (e, f: stress statistic = 0.245) in *Salmonella*-positive and *Salmonella*-negative animals on Rottnest Island. Covariates: season for left hand side plots, sex for right hand side plots. Key legends should be read for the left hand and right hand panels separately. Note that the distances along the axes are unit-less, therefore the positions of the points in the plots are relative distances from one another rather than absolute differences read in these units.
